# Prevalence and risk factors of type II endoleaks after endovascular aneurysm repair: A meta-analysis

**DOI:** 10.1371/journal.pone.0170600

**Published:** 2017-02-09

**Authors:** Qiang Guo, Xiaojiong Du, Jichun Zhao, Yukui Ma, Bin Huang, Ding Yuan, Yi Yang, Guojun Zeng, Fei Xiong

**Affiliations:** Department of Vascular Surgery, West China Hospital, Sichuan University, Chengdu, Sichuan Province, China; Medical University Innsbruck, AUSTRIA

## Abstract

**Objectives:**

This systematic review and meta-analysis aims to determine the current evidence on risk factors for type II endoleaks after endovascular aneurysm repair (EVAR).

**Materials and methods:**

A systematic literature search was carried out for studies that evaluated the association of demographic, co-morbidity, and other patient-determined factors with the onset of type II endoleaks. Pooled prevalence of type II endoleaks after EVAR was updated.

**Results:**

Among the 504 studies screened, 45 studies with a total of 36,588 participants were included in this review. The pooled prevalence of type II endoleaks after EVAR was 22% [95% confidence interval (CI), 19%–25%]. The main factors consistently associated with type II endoleaks included age [pooled odds ratio (OR), 0.37; 95% CI, 0.31–0.43; P<0.001], smoking (pooled OR, 0.71; 95% CI, 0.55–0.92; P<0.001), patent inferior mesenteric artery (pooled OR, 1.98; 95% CI, 1.06–3.71; P = 0.012), maximum aneurysm diameter (pooled OR, 0.23; 95% CI, 0.17–0.30; P<0.001), and number of patent lumbar arteries (pooled OR, 3.07; 95% CI, 2.81–3.33; P<0.001). Sex, diabetes, hypertension, anticoagulants, antiplatelet, hyperlipidemia, chronic renal insufficiency, types of graft material, and chronic obstructive pulmonary diseases (COPD) did not show any association with the onset of type II endoleaks.

**Conclusions:**

Clinicians can use the identified risk factors to detect and manage patients at risk of developing type II endoleaks after EVAR. However, further studies are needed to analyze a number of potential risk factors.

## Introduction

Endovascular aneurysm repair (EVAR) has become the primary choice of treatment for abdominal aortic aneurysms (AAAs) in suitable patients [1]. EVAR always has better short-term outcomes compared with open repair [2,3]. Aortic endograft occlusion, migration, and endoleaks are known complications after EVAR [4], among which endoleaks are the most frequent. Types I and III endoleaks require urgent intervention to relieve aneurysm re-pressurization [5,6]. Type II endoleaks are caused by backflow of collateral arteries into the aneurysm sac, with an occurrence rate of 10.2% after EVAR [7]. Type II endoleaks do not exert any immediate adverse effects. However, persistent type II endoleaks are believed to be associated with increased sac pressure and cause adverse outcomes and even aneurysm rupture [8].

Various studies assessed risk factors for type II endoleaks following EVAR [9–11]. Potential correlations may exist between preoperative characteristics and development of type II endoleaks [9]. A patent inferior mesenteric artery (IMA) and the number of patent lumbar arteries have been investigated as risk factors for type II endoleaks [10,11]. However, no previous systematic review has concentrated on this topic. Hence, the primary objective of the present systematic review and meta-analysis was to determine the current evidence on risk factors for the onset of type II endoleaks. This review concentrated on nonclinical risk factors, such as sex and age, preoperative comorbidities, and aortic anatomy. Moreover, we intended to report the updated pooled prevalence of type II endoleaks after EVAR.

## Methods

### Search strategy and study selection

Relevant publications were identified by searching the following database: MEDLINE (from Jan 1, 1950, to June 30, 2016) (S1 File), Scopus (from 1966 to June 30, 2016), and EMBASE (from 1966 to June 30, 2016). The terms “type II endoleak” or “type 2 endoleak,” “endovascular,” and “aneurysm” were searched. The references cited in published original and review articles were searched to identify additional studies. The search was limited to human adults and publications in English. Subsequently, two authors (Q.G. and X.D.) independently reviewed the title and abstract of the studies identified in the search to exclude those that did not answer the research question of interest in accordance with prespecified inclusion and exclusion criteria. Records extracted by the initial search were screened, and potentially relevant papers were retrieved and examined in detail. Duplicate publications and case reports were also excluded. Whenever publications of overlapping populations were available, the publication with the most complete and relevant set of data was chosen.

We identified studies in accordance with the following inclusion criteria: (1) participants: human adults (minimum of 18 years of age) with abdominal aortic aneurysm; (2) intervention: EVAR; (3) comparison: patients with potential risk factors versus patients without potential risk factors resulting in type II endoleaks; and (4) sufficient data were available for estimating an odds ratio (OR) with confidence interval (CI).

### Data abstraction and quality assessment

Two authors (B.H. and Y.M.) extracted relevant data independently, including the first author’s name, study year, study region, study design, number of type II endoleaks, number of reintervention for type II endoleaks, duration of follow-up, and potential risk factor for type II endoleaks [including age, sex, smoking, diabetes, hypertension, hyperlipidemia, chronic renal insufficiency, chronic obstructive pulmonary diseases (COPD), polytrafluoroethylene-based endografts, anticoagulants, antiplatelet, patent inferior mesenteric artery, maximum aneurysm diameter, and number of patent lumbar arteries]. Conflicts in data abstraction were resolved through consensus by referring back to the original article. The methodological quality of the studies was assessed by two authors (D.Y. and Y.Y.) independently using the Newcastle–Ottawa scale [12]. Quality was calculated on the basis of the following three aspects of study design: patient selection, comparability of study groups, and exposure and outcome of study participants. Studies that scored 5 or greater were considered high quality, whereas those that scored 4 and below were considered low quality.

### Statistical analysis

Statistical analyses were performed with Stata 12.0 software (StataCorp LP, College Station, Texas, USA). Dichotomous variables were analyzed by estimating the odds ratio (OR) with a 95% confidence interval (95% CI), whereas continuous variables were analyzed using the weighted mean difference with a 95% CI. Statistical heterogeneity between studies was assessed using the *I*^2^ measure. I^2^ > 50% was considered to indicate high statistical heterogeneity. If no heterogeneity was found, a fixed effect model based on the Mantel and Haenszel estimator was used; otherwise, a random effect model based on the DerSimonian and Laird estimator was used [13]. Several characteristics were identified to analyze their effect on the prevalence of type II endoleaks. Categorical characteristics were treated as moderators, and effectiveness was compared across subgroups formed by year of publication (before 2010 vs. 2011–2016), surveillance test (with categories: CTA vs. others), and study setting (retrospective vs. prospective). Egger’s and Begg’s tests were performed to assess publication bias [14]. For all statistical analyses, except heterogeneity and publication bias, p < 0.05 was regarded to indicate statistical significance, and all tests were two-sided. The present systematic review was conducted and reported in accordance with the guidelines of Preferred Reporting Items for Systematic Reviews and Meta-Analyses (S2 File) [15].

## Results

### Study characteristics and methodological quality

**Fig 1:**
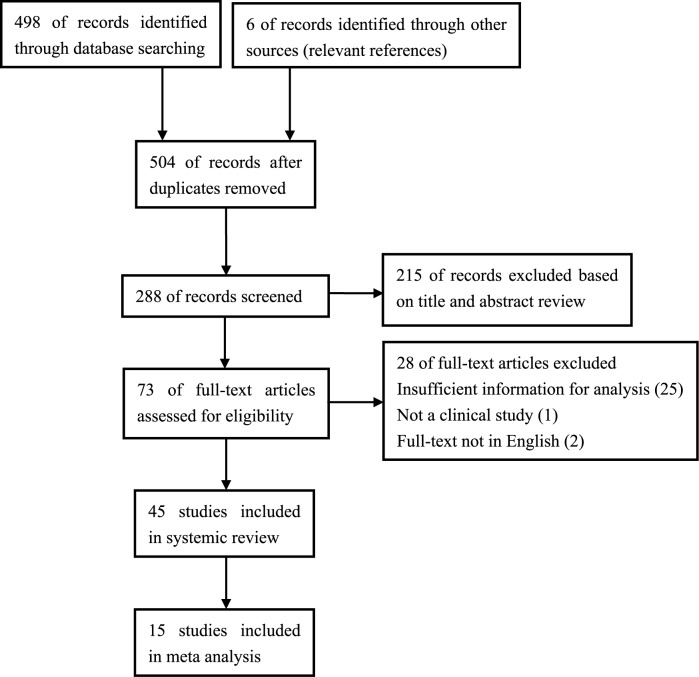
PRISMA flow chart for the review.

From the 504 studies identified using our search strategy, 73 potentially relevant articles were retrieved and assessed for eligibility. In total, 45 papers were included in this review [8–11,16–56], with 15 included in the meta-analysis [8,9,28,32–34,37,38,45–48,50,55,56]. The flow diagram summarizing the study identification and selection is shown in Fig 1. The characteristics of the included studies are reported in Table 1. The methodological quality of the included studies for meta-analysis is summarized in S3 File. All of the studies enrolled for meta-analysis were of high quality.

**Table 1:** Characteristics of included studies.

Studies	Country	Study design	No. of patients	Type II endoleaks	types of endoleaks	Reintervention	Follow-up (months)	Surveillance test
Chuter 2001[16]	USA	retrospective	114	9	Any	n.s.	n.s.	CTA
Haulon 2001[17]	France	retrospective	60	18	Any	n.s.	13.3	CTA/MRI
Liewald 2001[18]	Germany	retrospective	160	13	Any	n.s.	n.s.	CTA
Buth 2002[19]*	Netherland	retrospective	3529	240	Any	53	n.s.	CTA
Parent 2002[20]	USA	retrospective	83	36	Any	3	20.7	CTA
Solis 2002[21]	USA	retrospective	119	14	Any	10	18.2	CTA
Tuerff 2002[22]	USA	prospective	130	22	Any	n.s.	15.7	CTA
Faries 2003[23]	USA	retrospective	597	16	Any	n.s.	24.5	CTA
Farner 2003[24]	USA	retrospective	63	9	Any	n.s.	12	CTA
Kasirajan 2003[25]	USA	retrospective	104	8	Any	n.s.	n.s.	CTA
Carpenter 2004[26]	USA	prospective	227	21	Any	8	n.s.	CTA
Steinmetz 2004[27]	USA	retrospective	486	90	Any	4	21.7	CTA
Van Marrewijk 2004[28]*	Netherland	prospective	3595	320	Any	n.s.	15	CTA
Tolia 2005[29]	USA	retrospective	83	16	Any	2	30	CTA
Sheehan 2006[30]	USA	retrospective	1909	221	Any	101	36	CTA
Silverberg 2006[31]	USA	retrospective	965	154	Any	19	22	CTA
Jones 2007[8]	USA	retrospective	873	164	Any	16	38	CTA
Abularrage 2010[9]	USA	retrospective	832	136	Persistent	39	34.8	CTA
Brountzos 2012[10]	Greece	retrospective	136	31	Persistent	n.s.	13.5	CTA
Koole 2012[32]*	Netherland	prospective	8638	4518	Any	661	26	CTA
Nolz 2012[33]	Austria	retrospective	407	49	Any	20	n.s.	CTA
Batti 2013[34]	France	prospective	700	201	Any	30	31.3	CTA
Chang 2013[35]	USA	retrospective	1736	474	Any	251	32.4	CTA
Jouhannet 2013[36]	France	retrospective	232	94	Any	14	23	CTA
Cieri 2014[37]	Italy	prospective	1412	218	Any	52	45	CTA
Maeda 2014[11]	Japan	retrospective	469	111	Any	n.s.	21.0	CTA
Sidloff 2014[38]	UK	prospective	904	175	Any	9	43.2	DUS
Ward 2014[39]	USA	retrospective	326	99	Any	n.s.	n.s.	CTA
Dudeck 2015[40]	Germany	retrospective	188	62	Any	n.s.	39	CTA
Hiraoka 2015[41]	Japan	retrospective	148	36	Any	n.s.	28.8	CTA
Löwenthal 2015[42]	Germany	retrospective	130	62	Any	n.s.	22	CTA
Müller-Wille 2015[43]	Germany	retrospective	384	56	Any	20	36	CTA
Gallitto 2015[44]	Italy	prospective	200	47	Any	6	22	CTA
Kray 2015[45]	USA	retrospective	191	18	Any	n.s.	n.s.	CTA
Nolz 2015[46]	Austria	retrospective	143	69	Any	n.s.	n.s.	CTA
Pini 2015[47]	Italy	retrospective	753	85	Any	2	19	CTA/DUS
Walker 2015[48]	USA	retrospective	1736	474	Any	111	32.2	CTA
Rousié 2015[49]	Belgium	retrospective	77	17	Any	0	79	CTA
Phan 2015[50]	Germany	retrospective	82	51	Any	13	29.5	CTA
Piazza 2016[51]	USA	prospective	126	34	Any	7	16	CTA
Lo 2016[52]	USA	retrospective	2367	807	Persisent	n.s.	15.4	n.s.
Mursalin 2016[53]	Japan	retrospective	145	47	Any	n.s.	n.s.	CTA
Ikoma 2016[54]	Japan	prospective	34	12	Any	n.s.	n.s.	CTA
Pippin 2016[55]	USA	retrospective	163	66	Any	4	24.7	CTA
Fujimura 2016[56]	Japan	retrospective	832	234	Any	11	35.6	CTA/MRI

*Three papers were published from the EUROSTAR registry and may present duplicated data.

n.s., Not stated.

### Prevalence of type II endoleaks after EVAR

**Fig 2:**
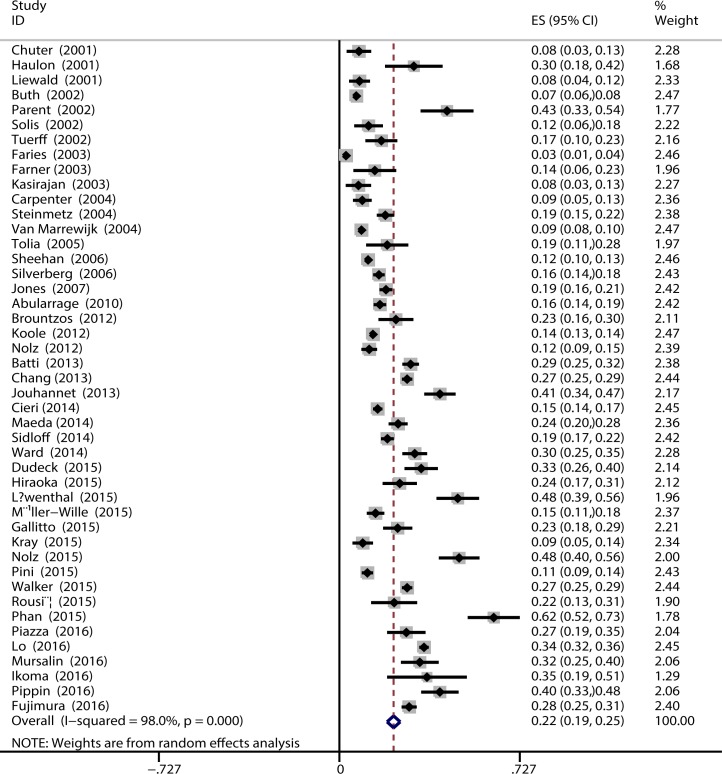
Pooled prevalence of type II endoleaks after EVAR.

A total of 9,654 type II endoleaks were reported to have occurred over a range of 12–79 follow-up months in 36,588 patients. The pooled prevalence of type II endoleaks after EVAR was 22% (95%CI, 19%–25%) (Fig 2). Reintervention for type II endoleaks was reported in 1466 (19%) of 7885 patients. The proportion of type II endoleaks was significantly greater in subgroups of studies published after 2010 [13%, 95% CI (11%–16%) vs. 27% 95% CI (24%–31%)] (Table 2). The proportion of type II endoleaks was similar in subgroups of studies using CTA as surveillance test with those using others [21%, 95% CI (14%–23%) vs. 24%, 95% CI (14%–34%)]. The proportion of type II endoleaks was similar in subgroups of prospective studies with retrospective studies [18%, 95% CI (15–21%) vs. 23%, 95% CI (19%–27%)].

**Table 2:** Subgroup analysis for prevalence of Type II endoleak.

Study subgroup	No. of studies	Total no. of participants	Pooled prevalence	95% CI	I^2^
Total	45	36588	0.22	0.19–0.25	98.0
Year of the study					
2001–2010	18	13929	0.13	0.11–0.16	95.1
2011–2016	27	22659	0.27	0.24–0.31	97.5
Study setting					
Prospective	10	15966	0.18	0.15–0.21	96.1
Retrospective	35	20622	0.23	0.19–0.27	98.3
Surveillance test					
CTA	40	31672	0.21	0.19–0.24	97.6
Others	5	4916	0.24	0.14–0.34	98.4

### Meta-analysis of possible factors associated with type II endoleak

In total, eight studies reported on the effect of age on type II endoleaks (Table 3). These studies consistently demonstrated that age was a risk factor for the onset of type II endoleaks, although considerable heterogeneity was present among the results reported (I^2^ = 99.0%). The pooled OR of the eight studies was 0.37 (95% CI, 0.31–0.43; P<0.001). Twelve studies investigated smoking as a risk factor for the onset of type II endoleaks. Among these 14 studies, a considerable heterogeneity was observed between the study findings (I^2^ = 86.4%); however, all studies were generally consistent in reporting smoking as a protective factor for the onset of type II endoleaks. The pooled OR of the 12 studies was 0.71 (95% CI, 0.55–0.92; P<0.001).

**Table 3:** Pooled ORs for association of commonly studied risk factors with Type II endoleak.

Potential risk factors	No. of studies	Total no. of participants	Pooled OR	95% CI	P value	I^2^
Age	8	6278	0.37	0.31–0.43	<0.001	99.0
Male	12	11775	0.83	0.67–1.02	0.059	46.4
Smoking	14	20477	0.71	0.55–0.92	<0.001	86.4
Diabetes	10	7303	0.91	0.76–1.09	0.251	20.9
Hypertension	10	7281	0.98	0.85–1.12	0.484	0
Hyperlipidemia	7	5522	1.12	0.83–1.49	0.814	74.7
Chronic renal insufficiency	10	9201	0.85	0.53–1.36	0.600	85.3
COPD	10	5745	0.84	0.69–1.03	0.135	34.1
Polytrafluoroethylene-based endografts	7	8396	0.88	0.65–1.18	0.390	70.8
Anticoagulants	5	3758	1.27	0.97–1.67	0.537	0
Antiplatelet	5	3758	1.09	0.79–1.51	0.220	65.6
Patent IMA	3	4353	1.98	1.06–3.71	0.012	77.6
Number of patent lumbar arteries	2	758	3.07	2.81–3.33	<0.001	99.8
Maximum aneurysm diameter	7	4858	0.23	0.17–0.30	<0.001	98.0

Seven cohort studies assessed the effect of maximum aneurysm diameter on the onset of type II endoleaks. The I^2^ among the seven studies was 98.0%, and the resulting pooled OR was 0.23 (CI, 0.17–0.30; P = 0.012), showing an increased risk of type II endoleaks in patients with a greater diameter of AAA. Three studies were included in the meta-analysis of patent IMA as a risk factor for the onset of type II endoleaks. The extent of heterogeneity present between the findings reported was considerable (I^2^, 77.6%), and the pooled OR was 1.98 (95% CI, 1.06–3.71; P<0.001). Two studies reported the effect of number of patent lumbar arteries on type II endoleaks, and the pooled OR was 3.07 (95% CI, 2.81–3.33; P<0.001).

Twelve studies assessed male sex as a potential risk factor. The pooled OR of 0.83 (95% CI, 0.67–1.02; I^2^, 46.4%) suggests that male sex is not associated with type II endoleaks. Diabetes (OR 0.91; 95% CI, 0.76–1.09), hypertension (OR, 0.98; 95% CI, 0.85–1.12), Polytrafluoroethylene-based endografts (OR, 0.88; 95% CI, 0.65–1.18), anticoagulants (OR, 1.27; 95% CI, 0.97–1.67), antiplatelet (OR, 1.09; 95% CI, 0.79–1.51), hyperlipidemia (OR, 1.12; 95% CI, 0.83–1.49), chronic renal insufficiency (OR, 0.85; 95% CI, 0.53–1.36), and COPD (OR, 0.84; 95% CI, 0.63–1.03) are not risk factors of type II endoleaks (Table 3).

Sensitivity analyses by quality (methodological quality >6), and sample size (n>100) were done for the enrolled studies. All meta-analytic conclusions remained robust to this testing. The results of Begg’s test (p = 0.432) and Egger’s test (p = 0.338) revealed no significant publication bias in this study.

## Discussion

Endoleaks are specific complications of EVAR. Type II endoleaks are the most common type of endoleaks, occurring in approximately 10% of patients after EVAR [7]. Although Type II endoleaks are less likely to require secondary reintervention than type I or III endoleaks because of the possibility of arterial rupture [9], type II endoleaks are a risk factor for aneurysm sac growth, and ruptures occur in approximately 1% of patients with type II endoleaks after EVAR [7]. Thus, patients need continuous surveillance after EVAR to detect aneurysm growth and endoleaks. Previous studies explored risk factors for type II endoleaks after EVAR [9–11]. However, controversies have been found between these studies. These discrepancies may be attributed to the insufficient statistical power of individual studies and the inability to perform separate analyses. Therefore, we conducted a systematic review and meta-analysis to identify the risk factors for type II endoleaks after EVAR. Furthermore, the pooled prevalence of type II endoleaks must be updated on the basis of the latest evidence.

Among the potential risk factors for type II endoleaks, older age, patent inferior mesenteric artery, number of patent lumbar arteries, and maximum aneurysm diameter significantly increase the risk for type II endoleaks after EVAR. By contrast, smoking is a protective factor for type II endoleaks. Sex, diabetes, hypertension, anticoagulants, hyperlipidemia, chronic renal insufficiency, COPD, and polytrafluoroethylene-based endografts are not associated with the onset of type II endoleaks. To the best of our knowledge, this systematic review and meta-analysis is the first to identify the risk factors of type II endoleaks after EVAR. This paper also revealed a higher prevalence of type II endoleaks after EVAR compared with an earlier review [7]. Most of the included studies exhibited high methodological quality on the basis of the Newcastle–Ottawa scale. Statistical tests were performed to examine the issue of publication bias, and results revealed no statistically significant publication bias.

Sidloff et al. [7] performed a systematic review that included 22 studies to evaluate the prevalence of type II endoleaks. A total of 1,515 type II endoleaks were reported to have occurred in 14,794 patients (10.2%). In this study, 9,654 of 36,588 patients developed type II endoleaks after 12–45 months of follow-up (26.4%). The proportion of type II endoleaks was significantly greater in the subgroup analysis of studies published after 2010 compared with those published before 2010 (27% vs. 13%). These results may be attributed to the improved accuracy of imaging tools for the surveillance after EVAR, more endoleaks might be detected by the upgraded imaging tools.

Reintervention for type II endoleaks has been reported in 1,466 (19%) of 7,885 patients. Management of type II endoleaks is still debated because clinical outcomes vary in published literature [34,36,37,44]. Some studies have indicated that persistence of type II endoleaks is not a negligible complication that could require re-interventions and decrease the long-term EVAR success [44]. Moreover, the endovascular reinterventions for type II endoleaks associated with an enlargement of the aneurysmal sac after EVAR have a poor effectiveness on the stabilization of the diameter of the AAA [36]. Other studies stated that recurrent and persistent type II endoleaks are prone to life-threatening complications, and type II endoleak appears to be a marker of EVAR failure that is difficult to predict and treat effectively [34,37]. The debate about the indication of intervention for type II endoleaks seems to last.

From the anatomic aspect, we identified patent IMA, number of patent lumbar arteries, and maximum aneurysm diameter as risk factors for the onset of type II endoleaks. Preoperative embolization of the IMA has been proved to be associated with decreased risk of type-2 endoleak and aneurysm sac enlargement after EVAR [57]. Considering aneurysm diameter, it is likely that the increased flow channel within the aneurysm sac coupled with a larger number of patent aortic branch vessels leads to increased flow velocities within the sac, thus decreasing the likelihood of type II endoleak resolution [58]. It is reported that the number of patent lumbar arteries was also correlated with onset of type II endoleak, however, this study did not include the analysis because no other studies reported the similar issue [9].

In total, 12 studies investigated smoking as a risk factor for the onset of type II endoleaks. All studies were generally consistent in reporting smoking as a protective factor for the onset of type II endoleaks. Koole et al. [32] reported a study that analyzed the data of 8638 patients who were enrolled prospectively in the European Collaborators on Stent-Graft Techniques for Aortic Aneurysm Repair database. The study observed decreased intraoperative perfusion from the mesenteric inferior artery or lumbar arteries in smokers. The explanation of these findings remains unclear; however, we hypothesized that tobacco use can induce the atherosclerotic injury of medium- and small-sized vessels, which may narrow or occlude these arteries and thereby impair the blood flow [32]. There is also an increased tendency of coagulation in smokers, which also might narrow or occlude the arteries. We also found that older age is a risk factor for the onset of type II endoleaks. Long history and large diameter of AAA in aging patients, favoring high-pressure aneurysm sac, may be a possible explanation of this fact. Besides, aging patients might also have more severe atherosclerotic injury of vessels which induced impair of the retrograde flow into abdominal aorta.

Our review also has some limitations. First, only the main predictors drawn from the articles were analyzed because of data deficiencies. Second, our study was subjected to heterogeneity in some of the inclusion criteria, as well as relatively small sample sizes and numbers of articles, which may have caused potential publication bias. Only three studies were included in the meta-analysis of patent IMA as a risk factor for the onset of type II endoleaks, whereas only four studies provided data that compared the effects of anticoagulants used on the onset of type II endoleaks. Furthermore, most of the articles included in our review are retrospective observational studies. A long-term prospective study to specifically define risk factors of endoleaks would be instructive.

In conclusion, age, patent inferior mesenteric artery, number of patent lumbar arteries, maximum aneurysm diameter, and non-tobacco use are significant factors in predicting type II endoleaks. Meanwhile, sex, diabetes, hypertension, anticoagulants, antiplatelet, hyperlipidemia, chronic renal insufficiency, types of graft material, and COPD show no statistical significance. Thus, clinicians should pay attention to these factors in AAA patients after EVAR.

## Supporting information

S1 File Medline search strategy. (DOC)

S2 File PRISMA 2009 checklist. (DOC)

S3 File Quality assessment of included studies. (DOC)
