# Supplementary material for: Prevalence and risk factors of type II endoleaks after endovascular aneurysm repair: A meta-analysis
Source: PLoS One. 2017 Feb 9;12(2):e0170600. doi: 10.1371/journal.pone.0170600 (PMC5300210; doi:10.1371/journal.pone.0170600)
Supplement: S1 File — (DOC) [file pone.0170600.s001.doc]

**S1: Medline search strategy**

#1 exp type II endoleaks/

#2 (type II endoleak or type 2 endoleak or type II leak or type 2 leak).tw

#3 1 or 2

#4 exp EVARs/

#5 (endovascular abdominal aortic aneurysm repair or EVAR or endovascular repair or endovascular or stentgraft).tw

#6 4 or 5

#7 exp AAAs/

#8 (abdominal aortic aneurysm or AAA or aneurysm).tw

#9 7or 8

#10 3 and 6 and 9
