# Supplementary material for: Prevalence and risk factors of type II endoleaks after endovascular aneurysm repair: A meta-analysis
Source: PLoS One. 2017 Feb 9;12(2):e0170600. doi: 10.1371/journal.pone.0170600 (PMC5300210; doi:10.1371/journal.pone.0170600)
Supplement: S3 File — (DOC) [file pone.0170600.s003.doc]

**S3. The quality assessment of individual studies included for meta analysis.**

| Study | Selection | Comparability | Outcome | Total |
| --- | --- | --- | --- | --- |
| Abularrage 2010 | 4 | 2 | 2 | 8 |
| Batti 2013 | 4 | 2 | 2 | 8 |
| Cieri 2014 | 4 | 2 | 2 | 8 |
| Fujimura 2016 | 4 | 2 | 2 | 8 |
| Jones 2007 | 4 | 2 | 2 | 8 |
| Koole 2012 | 4 | 0 | 2 | 6 |
| Kray 2015 | 4 | 2 | 2 | 8 |
| Nolz 2012 | 4 | 0 | 2 | 6 |
| Nolz 2015 | 4 | 2 | 3 | 9 |
| Pini 2015 | 4 | 2 | 2 | 8 |
| Phan 2015 | 4 | 2 | 2 | 8 |
| Pippin 2016 | 4 | 2 | 2 | 8 |
| Sidloff 2014 | 4 | 2 | 2 | 8 |
| van Marrewijk 2004 | 4 | 2 | 2 | 8 |
| Walker 2015 | 4 | 2 | 3 | 9 |
